# Imaging Studies in Focal Dystonias: A Systems Level Approach to Studying a Systems Level Disorder

**DOI:** 10.2174/157015913804999513

**Published:** 2013-01

**Authors:** Anne J Blood

**Affiliations:** Mood and Motor Control Laboratory, Laboratory of Neuroimaging and Genetics, Athinoula A. Martinos Center for Biomedical Imaging, Charlestown, MA, Departments of Psychiatry and Neurology, Massachusetts General Hospital, Harvard Medical School, Boston, MA

**Keywords:** Dystonia, fMRI, DTI, PET, TMS, MEG, posture, basal ganglia, premotor, cerebellum, botulinum toxin, DBS.

## Abstract

Focal dystonias are dystonias that affect one part of the body, and are sometimes task-specific. Brain imaging and transcranial magnetic stimulation techniques have been valuable in defining the pathophysiology of dystonias in general, and are particularly amenable to studying focal dystonias. Over the past few years, several common themes have emerged in the imaging literature, and this review summarizes these findings and suggests some ways in which these distinct themes might all point to one common systems-level mechanism for dystonia. These themes include (1) the role of premotor regions in focal dystonia, (2) the role of the sensory system and sensorimotor integration in focal dystonia, (3) the role of decreased inhibition/increased excitation in focal dystonia, and (4) the role of brain imaging in evaluating and guiding treatment of focal dystonias. The data across these themes, together with the features of dystonia itself, are consistent with a hypothesis that all dystonias reflect excessive output of postural control/stabilization systems in the brain, and that the mechanisms for dystonia reflect amplification of an existing functional system, rather than recruitment of the wrong motor programs. Imaging is currently being used to test treatment effectiveness, and to visually guide treatment of dystonia, such as placement of deep brain stimulation electrodes. In the future, it is hoped that imaging may be used to individualize treatments across behavioral, pharmacologic, and surgical domains, thus optimizing both the speed and effectiveness of treatment for any given individual with focal dystonia.

## INTRODUCTION

Focal dystonias affect one part of the body, most commonly the neck (cervical dystonia), hand (focal hand dystonia), laryngeal muscles (spasmodic dysphonia), eyelids (blepharospasm), and mouth (oromandibular dystonia). Studies estimate that there are between 30 and 7320 cases per million of primary late onset primary dystonia, which includes focal, limb, and segmental forms, but this is likely to be an underestimate [1]. Some focal dystonias are task-specific, such that dystonic symptoms are only observed during certain motor tasks. Task-specific dystonias are sometimes seen in musicians and other individuals who perform repetitive motor tasks. However, just as often, task-specific focal dystonias are observed in populations who are not exposed to repetitive tasks; an example of this is spasmodic dysphonia, in which the laryngeal muscles contract excessively during speech. Other focal dystonias are present at rest and/or when the individual is in certain postural positions, such as sitting, standing upright, or walking (versus lying down); the effect of body position on focal dystonia symptoms is a frequent factor in the expression of some cervical dystonia symptoms. Thus, focal dystonia does not specify etiology, it is a classification based on localization of symptoms. However, focal dystonias are more likely to develop in adulthood than in childhood, and are less likely to be associated with known genetic bases for dystonia, although early onset and genetic forms of focal dystonia are known to occur [2].

Individuals with task-specific focal dystonias can more easily be studied in dystonic versus non-dystonic states than other dystonias, and thus such patients are frequently used to conduct fMRI studies. However, the literature is not limited to these forms of dystonia, and there is a broad range of structural and functional studies of focal dystonias of all kinds. Over the past 30 years, brain imaging and transcranial magnetic stimulation (TMS) methods have been critical in showing that individuals with focal dystonias exhibit abnormal brain function, and in some cases structure or connectivity, across a broad set of brain sensorimotor regions (reviewed extensively elsewhere, e.g., [3-9]). In the current review article, we will discuss how recent imaging studies advance our understanding of the pathophysiology and treatment of focal dystonia, focusing on the prominent themes in recent literature, and discuss how the collective findings support some more general principles about dystonia. Specifically, we will focus on (1) recent findings of abnormal function/structure in premotor brain regions and the cerebellum in focal dystonia, and clues to what "functional" system is affected in dystonia, (2) the proposal that sensorimotor integration is impaired in dystonia, (3) the role of impaired inhibition/selectivity and reorganization of the motor system in dystonia, and (4) using imaging to guide and test effectiveness of treatment in focal dystonia. Each section will discuss what insights the topic has provided about mechanisms of dystonia. One important issue that will be addressed is that (1) – (3) typically are treated as discrete issues in the literature, without suggestion of how they relate to each other, or what they have in common in relation to dystonia. One potential explanation for the relationship between them will be offered here, based on the author’s own work. 

What is the general benefit of using imaging to study focal dystonias? Imaging provides clues to the circuitry involved in dystonia, including its direct relationship to symptoms, and “endophenotypic” traits that appear to be observed in the absence of symptoms. It also allows us to test how effective treatments change the brain so we better understand their physiological basis. Finally, imaging has the potential to provide clues about predicting which treatment(s) will most benefit a given dystonia patient.

## IMAGING AND THE IMPORTANCE OF MODELS FOR FOCAL DYSTONIAS

Because imaging is a systems-level technique, imaging studies should give us clues about a systems level interpretation of the disorder being studied, as well as allowing us to test existing models. Most existing models for dystonia point to strong evidence for insufficient inhibition of motor programs [3, 10-14]. Task specific focal dystonias are most commonly thought of as inappropriate activation of motor programs that "compete" with the desired movement [12], and the physiologic mechanism is thought in some cases to reflect issues with "surround" inhibition in motor cortical regions and/or basal ganglia regions [13]. While this general model is very consistent with empirical evidence in dystonia, the functional interpretation of these findings, and the way in which they tie into other abnormalities observed in focal dystonias, has remained more elusive. Currently, the collection of diverse findings in the dystonia imaging literature tends to be interpreted as a collection of diverse mechanisms for dystonia, including motor planning abnormalities and impaired sensorimotor integration, suggesting a higher order and more complex mechanism for dystonia than a simple shortage of inhibitory control. Moreover, most of the proposed mechanisms are attributed to errors or disorganization in the computation of motor programs. A challenge to these interpretations is a missing explanation how errors and/or disorganization would lead to the stereotyped symptoms we see in dystonia specifically, as opposed to other motor abnormalities.

An alternative interpretation of dystonia has been proposed, which suggests we have the intuition to call all dystonias by the same name because we recognize that they do indeed have something in common, but whatever it is does not fit into a single neural functional system *as currently defined*. Specifically, this conceptual model hypothesizes that all dystonias reflect excessive amplification of at least one component of a general neural postural control/stabilization system, including any component that provides a stabilizing force within the body or to a movement, either globally (e.g. balance, control of body position and movements in space) or locally (e.g. laryngeal position resisting airflow to produce speech, control of eyelid and jaw position) [15]; features of this model are summarized in Fig. (**1**). This system would include a number of subcomponents, which would each be activated under different circumstances (including balance, reflexes, body position at rest and during movement, and a “control” component for movements themselves, discussed in the next paragraph), and exhibit different qualitative features, including neural programs that are static versus dynamic. Thus, this functional system would not be limited to “position”, which is the most standard definition of posture. Rather, this functional system would include both, what is thought of as traditional postural function, and other functions not typically thought of as posture, including components of fine motor control.

In particular, this model suggests how we can relate task-specific dystonias—which are thought to be disorders of fine motor control [4, 11, 16] rather than posture—to other focal, and even generalized dystonias. The model proposes that motor control is implemented using two parallel functional systems, one that selects master motor programs and another that acts as a "controller" of the movement using both feedforward and feedback mechanisms [15] (Fig. (**1A,C**)), analogous to the navigation versus stabilization systems of an airplane or motor vehicle. This "controller" is proposed to modulate precision, velocity, “stiffness”, and force of any given movement, and is a subcomponent of the postural control/stabilization system. Although it could be argued this is somewhat a semantic distinction in relation to the definition of fine motor control, this “dual-channel hypothesis” argues that the dystonic motor programs in task-specific dystonias are not recruited inappropriately, only at an inappropriate magnitude [15], whereas a “single-channel hypothesis” would be consistent with task-specific focal dystonias representing inappropriate activation of competing motor programs [12]. In addition, the “dual-channel hypothesis” argues that motor control mechanisms are overactive in dystonia (albeit not in a way that improves performance), rather than that motor control is lost. This dual-channel hypothesis was initially conceived during interpretation of diffusion tensor imaging data in dystonia patients (i.e., [17]), demonstrating an example of the hypothesis-generating capacity of imaging methods (see Fig. (**2**)).

What is the rationale for arguing that these postural control/stabilization functions are grouped together into a single brain functional system? Below, are two critical pieces of evidence:

1. A common set of mechanisms proposed for the implementation of postural control/stabilization function provides a simple and highly efficient means by which the brain could implement the many aspects of motor control, allowing an infinite number of ways to execute any single stored motor program, and allowing for adaptation and flexibility of that program. Specifically, all functions falling into the category of postural control and stabilization could be implemented by one of the following mechanisms for mechanical stabilization, and would result in an apparent "overflow" (i.e. look like dystonia) if amplified sufficiently outside the normal range: (1) cocontraction of antagonistic muscles, (2) recruitment of muscles antagonistic to a movement, or (3) recruitment of synergistic groups of agonist muscles; the general tendency of these mechanisms to recruit *groups* of muscles is denoted in Fig. (**1C**) by showing output to more than one muscle for "postural" channels. These mechanisms would be highly subtle in the healthy individual (i.e. of nearly negligible amplitude), and scaled in accordance with the desired/required degree of control. Such mechanisms would also have a net (mechanical) inhibitory effect on movement, whether during healthy or disordered movement, suggesting a potential role for the indirect pathway in gating these functions since this pathway is thought to inhibit movement [18]. Though this would lead to a slight reduction in the efficiency of energy expenditure at the level of motor output itself, the resulting reduction in computational power and complexity of coding motor control, and the redundancy it would provide for body stability, would most certainly justify this energy trade-off.
  Based on the description of the mechanisms above, it can be seen that this system is actually best characterized by the functions it performs, rather than by subjective descriptions of behavior in which we see its manifestations (e.g. posture). The mechanisms described above can each be applied as functions that act like a resistor to gravity at rest or during static postures (generating a range of levels of body “stiffness”, depending on expected external deformation forces), and a dynamic resistor (somewhat like impedance, with the "movement" programs being the force vector, and the net velocity vector resulting from the movement/resistance ratio) during movement, serving the general purpose of mechanical control of the body. While for the purposes of the current paper we will refer to this as a postural control/stabilization system, note that this does not completely capture the full range of functions that the system is proposed to perform.
2. The basal ganglia neural architecture is consistent with two channels for motor control (Fig. (**1A**)), one of which coordinates across extensive regions/functions. Specifically, there exist two major subtypes of motor pallidal output neurons [19], one that collateralizes only minimally, which would be optimal for gating selection of specific, desired movement programs (e.g. voluntary movement), and another that exhibits extensive collateralization to the brainstem and within the thalamus (type Ib) (see Fig. (**1**), bottom left inset). It is proposed that the extensive arborization of this latter subtype is optimally suited to gate and coordinate across the anatomically and functionally broad range of posture and stabilization programs that would be recruited in parallel with movement. In addition to coordinating posture/stabilization programs that are activated simultaneously during movement, it is proposed that such architecture would allow for an efficient, toggle-like gating system between movement- and rest-related postural programs (see [15]; Fig. (**1B**)). It is not yet known if these axonal subtypes are distinctly associated with the direct versus indirect pathways of the basal ganglia, but this is an important future question to pose.
  As part of this review we discuss how this recently proposed model is consistent with each component of the imaging literature.

## DYSTONIA APPEARS TO BE A CIRCUIT DISORDER: WHAT IS THE FUNCTION OF THE CIRCUITS INVOLVED AND WHAT DO RECENT FINDINGS IN PREMOTOR AND CEREBELLAR REGIONS TELL US?

Because every functional imaging study conducted in dystonia has found altered activity levels across a number of motor and sensory regions in the brain, dystonia as a whole tends to be thought of as a "circuit" disorder. While the evidence for this is still indirect in that most imaging studies identify abnormalities in distinct brain regions and do not evaluate direct interactions between these regions, a few recent studies have begun evaluating the “network” aspect of these abnormalities. In this section we will first discuss findings in specific regions recently highlighted in the literature, and then discuss some studies evaluating the network as a whole.

Recently, there have been a number of studies implicating premotor cortical and cerebellar regions in dystonia. Below, these findings and their potential relevance are summarized.

### Premotor Abnormalities

There have been a number of recent studies focusing on abnormalities in premotor regions (dorsolateral premotor cortex and supplementary motor area) in focal dystonias, showing abnormal function in these regions either while moving, or during imagined movement. For example, studies have shown that premotor activity was abnormal in writer's cramp patients during imagined writing [20] and imagined drawing [21]. During imagined writing, premotor activity was found to be elevated in patients [20], while during imagined drawing, it was reduced (see Fig. (**3A**)) [21]. A study in cervical dystonia patients showed reduced activation in premotor cortex during imagined hand flexion and extension [22], while another study showed that treatment of cervical dystonia symptoms with botulinum toxin reduced SMA and dorsal premotor cortex activation during a skilled finger movement task (see Fig. (**3B**)) [23]. Premotor cortex has also been used as the target for successful treatment of cervical dystonia using either TMS or epidural stimulation (see Fig. (**3C**)) [24-26] (see Using imaging to evaluate and guide treatment of dystonia). Finally, premotor activation abnormalities (reduced activation) have also been shown in spasmodic dysphonia, during vocalization [27]. Many other studies have shown dorsal premotor and SMA activity abnormalities during movement tasks in focal dystonias, as far back as 1995 [28, 29], and in a handful of recent studies (e.g., [30-32]). Such abnormalities are not limited to focal dystonias, as they are observed in genetic forms of generalized dystonia as well (e.g., [33]).

Abnormalities in premotor function in focal dystonia tend to be interpreted as cognitive or computational errors at the planning stage of movement in dystonia. However, an alternative interpretation is that these findings actually reflect abnormal motor output [15]. Specifically, during a period of intention to move, it is known that there exist anticipatory postural adjustments (APAs; [34-37]), which consist of muscle activation that occurs at least 100 ms before an intended movement [37], and which are typically implemented with a large degree of muscle co-contraction [38]. APAs typically take place in the trunk and leg muscles when a global movement is being made [37], but also occur in the neck [38], shoulder, and elbow [39], particularly for tasks performed with the hand. Thus it is equally possible that dystonias that take place during preparation for movement reflect an upregulation of APAs [15]. In fact, it has been proposed that the SMA plays a role in postural control [40, 41], including potentially playing a role in anticipatory postural adjustments [41] and/or coordination between posture and movement [42]. At least one study has suggested that APAs take place during motor imagery [43], indicating the possibility that increased premotor function in dystonia patients in the motor imagery studies above reflected an increase in APAs. 

A similar argument holds true for several studies showing premotor (or other) abnormalities in dystonia during passive movement of a non-dystonic body region or during tasks that don't directly involve an individual moving a dystonic body part. Pelosin and colleagues [44] showed that patients with cervical dystonia showed abnormal trajectories of aimed movements of the arm, and that these trajectories improved after treatment with botulinum toxin. In addition, patients with cervical dystonia have been shown to exhibit abnormally increased fMRI activation in sensorimotor regions (contralateral primary and secondary sensory cortex, and cerebellum) during passive movement of the left forearm [45]. While it is quite possible (and indeed likely) that some of these abnormalities are endophenotypes and/or subclinical abnormalities that exist in focal dystonia, it must also be considered that axial (i.e. including the cervical region) body regions will recruit compensatory postural programs during movements of the arm (compensatory postural adjustments, or CPAs; [34, 36, 46, 47]). Without EMG measures of the cervical muscles, there is no way to verify an absence of abnormal muscle activity in the dystonic region secondary to the manipulation or movement of another region.

What forms of focal dystonia might reflect amplification of APAs? One example is writer’s cramp that involves shoulder and elbow elevation, and particularly individuals who exhibit these symptoms when merely picking up a pen. Cervical dystonias that become worse when the individual performs tasks that require increased head stabilization (e.g. when leaning forward) may also involve amplification of APAs. In contrast, dystonias reflecting amplification of CPAs would be ones which take place in reaction to demands on balance, such as when standing or walking, as compared to lying down. Cervical dystonias that improve in a supine position, and those with an effective sensory trick or "geste antagoniste" may involve pathophysiology in CPAs. Such forms of dystonia are likely to be more dynamic in nature than those involving amplification of APAs, since postural compensation tends to recruit dynamic programs that constantly adjust as the body moves, while APAs are implemented by more static stabilization of the body [48]. In the future, hypotheses relating to the role of postural function in dystonia can be tested more directly by evaluating APAs and/or CPAs in the patients described above, in conjunction with measurements of premotor function, and relating the magnitude of APAs/CPAs to the severity of dystonia.

### Cerebellar Abnormalities

It has been thought for some time that the cerebellum plays a role in at least some forms of dystonia, particularly because of animal models of dystonia that can be created or treated with alterations to the cerebellum [49], and because the basal ganglia and cerebellum are connected [50, 51]. Recently, a diffusion tensor imaging (DTI) study showed evidence for abnormalities in white matter projecting between the cerebellum and thalamus in patients with DYT6 dystonia (see Fig. (**4A**)) [52], which is the best characterized genetic form of dystonia that presents focally or multifocally and presents most commonly with craniocervical symptoms [53], although it can also become generalized [54]. Another DTI study in cervical dystonia patients without a known genetic basis, showed evidence for abnormal white matter connectivity between the pallidum and the red nucleus, which is a known relay nucleus for afferent connections to the cerebellum (see Fig. (**4B**)), and between the pallidum and the pedunculopontine nucleus [55]. Structural (gray matter density) and functional abnormalities in the cerebellum also have been shown in cervical dystonia [56], spasmodic dysphonia (e.g., [57, 58], see Fig. (**4C**)), and hand dystonias (e.g., [31, 32, 59]), as well as in generalized dystonias (e.g., [33, 52]). 

In the attempt to synthesize the information about circuitry involved in dystonia, the question arises whether these regions have anything in common with premotor regions, from a functional perspective, and what clues this gives us about what all dystonias have in common. Among the many functions attributed to the cerebellum, it is known to play a role in postural control, particularly postural control related to balance [60-62]. It is thought that the cerebellar vermis, in particular, plays a role in modulating upright stance and balance [63, 64]. Given that cervical dystonia affects proximal/axial muscles involved in balance, this link to the cerebellum and its role in balance seems especially intriguing. Given the proposed broadened definition of a more global posture/stabilization system above, it is possible that other regions of the cerebellum also mediate functions that are affected in dystonia. In addition, given the increasing evidence that the sensorimotor circuitry is extensively interconnected [65], including a recent study showing that the cerebellum is connected to premotor regions *via *the pons [64], it is difficult to know whether the cerebellum and premotor regions encode distinct subcomponents of postural control [15], or if they somehow generate postural control together. One might hypothesize that the cortical regions would be more likely to encode the "planning" aspect of postural control (i.e. APAs), while the cerebellum deals with more reflex-like functions (i.e. CPAs), but it is equally likely that they each contribute in different ways to both forms of postural control.

Given the extensive anatomical interconnections in the sensorimotor system, the concept of network interactions and their relevance to dystonia has gained recent interest, and can potentially tell us more about whether dystonia is related to impaired interactions between sensorimotor regions. Two recent EEG studies have used mutual information analysis and graph theoretic measures to evaluate functional connectivity within and across the sensorimotor network in focal hand dystonia patients, at rest and during a simple motor task that did not induce dystonic symptoms. The first study showed reduced mutual information in the beta band across cortical sensorimotor regions (as well as all regions evaluated) in patients with focal hand dystonia during the motor task [66]. The second study showed reduced efficiency of small-world sensorimotor networks in the beta band in focal hand dystonia patients during the motor task, relative to rest, whereas controls showed increased efficiency during the task [67]. Efficiency measures correlated inversely with the duration of the disease in patients. 

These studies clearly point to differences in the pattern of function between sensory and motor regions, relative to what is observed in healthy controls. The exact nature of these differences, however, is not yet clear. One interpretation of these results is that they reflect deficient brain connectivity between sensorimotor regions in focal hand dystonia patients [66, 67]. However, given that there is as of yet no direct evidence that functional connectivity analyses are indicative of information about structural connectivity [68], an alternative explanation is that differences in physiology across regions affect their functional synchronization. For example, increased gain between regions would potentially have an effect on mutual information between those regions, particularly if it is not evenly distributed across a given region or if it is threshold dependent, i.e. not always present. Mutual information between regions might also be lower if there were reduced function or number of inhibitory interneurons in these patients (for which we have evidence, e.g., [13]), since these neurons appear to play a role in modulating the synchrony of neural oscillations across the brain [69]. The finding of reduced efficiency in small-world networks is also interesting in light of the suggestion above that the postural control/stabilization system may accept a minor reduction in motor output efficiency to gain efficiency in coding motor control; such motor output efficiency and its neural correlates would be even further reduced in task-specific dystonias during movement, potentially even when symptoms are not present, as well as when they are. Such general “inefficiency” of motor output in dystonia may underlie the abnormalities that are observed even in the absence of dystonic symptoms [31, 44, 45, 66, 67].

## THE THEORY OF SENSORIMOTOR INTEGRATION ABNORMALITIES IN DYSTONIA

A number of studies have suggested that some forms of focal dystonia, particularly task-specific dystonias may at least partially result from disturbances in sensory function (reviewed extensively elsewhere, e.g., [70]) and, in particular, problems with sensorimotor integration [31, 71-75]. The therapeutic implications of these findings are significant in that they suggest why therapies that promote reorganization of sensory regions, including constraint-induced movement therapy [76], gradual re-learning of tasks by breaking them down into components that are first practiced in a non-target task context [77], proprioceptive training during which subjects distinguish between different frequencies of vibration [78], and learning to read Braille to increase sensory discrimination [79, 80], can sometimes be effective in treating dystonic symptoms. The identification of abnormalities in the sensory system may also be one of the most critical clues to determining the functional neural system that is affected in dystonia; this is discussed more below.

There have been a number of recent studies which have continued to add evidence to the role of the sensory system and sensorimotor integration in dystonia. These studies include identification of abnormal sensory activation in writer’s cramp [81], musician’s dystonias (e.g., [82]), and spasmodic dysphonia [57]. Abnormalities manifest as increased sensory function in some studies, while other studies show decreased sensory function, but overall it is agreed that there is altered representation of the affected region in some form, and often for other body regions as well [45, 83].

The general term “sensorimotor integration” refers to the communication between the sensory and motor regions, and the translation of the sensory “state” into the appropriate motor output [84]. The combination of abnormal sensory function and altered motor output in dystonia does indeed appear to indicate that what the motor cortex does in response to sensory information is different from that of healthy controls. The functional connectivity studies discussed above [66, 67], in particular, support a disruption in the synchrony of physiology between sensory and motor regions. However, the challenge is in defining what exactly does go wrong in the translation of sensory to motor function in dystonia. Is it that (1) the information does not transmit from sensory to motor regions to begin with? Or that (2) the sensory regions send wrong information to motor regions? Or finally, (3) is the correct sensory information sent, but the motor regions respond to the information incorrectly (e.g. respond to it at too great an amplitude)? Without an explicit answer to this question, the concept of sensorimotor integration does not provide an actual mechanism by which dystonic symptoms (as opposed to other motor abnormalities) are produced (having features of co-contraction and overflow, for example).

The third possibility seems most likely in the case of dystonia, since neither a lack of transmission nor an incorrect sensory signal (which could be incorrect in a multitude of ways) would be likely to lead to stereotyped symptoms. The critical role of the sensory system in dystonia, both in terms of association with symptoms and in alleviating symptoms [85], is consistent with the suggestion that dystonia involves abnormal amplification of the postural control/stabilization system: Since sensory information strongly drives recruitment of postural programs, the sensory system would be expected to be integral in driving dystonic symptoms if dystonia were an overactivation of postural programs. There need not be an error in sensory information to drive the dystonia, and there need not be an error in the communication between the sensory and motor systems (i.e., the sensory system need not recruit the *wrong* motor program); the relevant sensory input need only recruit the affected postural program. Over time, however, with the reinforcement of altered body positions, sensory representations would inevitably be changed, as has been shown with other long-term positional alterations [86]. With any increased representation that occurs during remapping, the threshold for recruiting the faulty motor program would be reduced, simply because the sensory region would be driven more strongly and/or at a lower threshold. And thus, there would be an associated lower threshold for recruiting dystonic programs. In sum, if there is an error at all in sensorimotor integration, it seems most likely to be increased gain from the sensory to the motor system [87].

What mechanism(s) might lead to amplification of the relevant motor programs? Given the known etiologic heterogeneity of dystonias, there are likely a number of answers to this question. A few possibilities, based on known physiologic and/or pharmacologic mechanisms, are suggested below, simply to convey feasibility of the systems-level findings with lower levels of neuroscience, but evidence for which, overall, is largely lacking in dystonia. Amplification of a posture/stabilization program could potentially result from changes in neurotransmitter and/or receptor function, particularly dopamine [88-90] and GABA [91]; in addition to a known association between reduced dopamine synthesis and dystonia [89], receptor density and function are known to change (and in particular, to upregulate) in animal models of Parkinson's Disease following structural deafferentation or neurotransmitter reduction [92-94]. Given that altered D2/D3 receptor binding has been shown in focal dystonias [95, 96], altered receptor function seems one possible candidate for changing the amplitude or gain of the relevant motor program. There is even initial evidence that the receptor subtype affected may determine which type of dystonia manifests [96]. Amplitude changes in postural control/stabilization programs may also result from brain plasticity impacting the strength of representation of a given motor program, either facilitated by neurotransmitter changes [97], or in association with excessive use of a motor program, such as in musician’s dystonia. The topic of brain plasticity in focal dystonia has been reviewed previously in a number of publications (e.g., [98-101]). Finally, there may be increased excitability of the neurons themselves in the relevant motor programs, whether due to axon physiology or to reduced afferent inhibition. Whatever the underlying etiology, there has been evidence for impaired inhibition and/or increased excitability in focal dystonias for some time, and recently, there has been a great deal of progress in this area. The next section addresses this topic, primarily using TMS methods.

## IMPAIRED INHIBITION/SELECTIVITY IN DYSTONIA

Beginning with TMS studies some years ago [102], investigators first began exploring a concept that fits well with the excessive motor output in dystonia: reduced inhibition and/or increased excitability in motor regions. Evidence for this has continued to build, including evaluations of this phenomenon in a number of motor regions. In these studies, typically, a conditioning pulse is made, and then a second pulse is delivered while motor evoked potentials (MEPs) are measured. In the past, studies have shown the basic principle of reduced surround inhibition (SI) in the motor cortex of individuals with focal dystonias (e.g., [13]), and have been reviewed elsewhere [6, 10, 103, 104]. SI is a phenomenon that has been observed in multiple brain regions, the most well-known as a mechanism in visual cortex to mediate visual contrast. In dystonia, SI has been proposed to serve the function of inhibiting competing movements while a particular movement is selected (e.g., [12]), thus focusing movement. Another possibility is that what appears to be SI, as measured by relative MEP amplitude at rest versus during a motor task, reflects the differential between (1) interneuron activity exerting inhibition of movement-related programs at rest in M1, and (2) activation of these programs during movement; the activity of inhibitory function at rest in healthy individuals could conceivably outweigh the activity relating to selective activation of motor programs during movement, since inhibitory interneuron function is thought to be a significant contributor to MEPs, particularly in response to repetitive TMS (rTMS) [105]. If inhibitory interneuron function in M1 were to be impaired in focal dystonia, this differential would be expected to be either inverted or missing, since there would be both, fewer inhibitory interneurons active at rest, and higher amplitude activation of motor programs during movement.

Abnormalities in intracortical inhibition (ICI) and other cortico-cortical interactions have been shown in individuals with focal dystonia (e.g., [106, 107]), and recently, studies have begun to investigate the relationship of these interactions to SI in motor cortex, to evaluate whether ICI may be the source of SI, and whether impairments in ICI lead to impaired SI in focal dystonia patients. Both the dorsal premotor (dPM) [108] and ventral premotor (vPM) [109] cortical projections to primary motor cortex (M1) have been tested as potential sources of SI. Although dPM did not appear to modulate SI in control subjects, it did appear to contribute to increased SI at rest and reduced SI during movement in focal hand dystonia patients (see Fig. (**3D**)) [108], while ventral premotor cortex (vPM) did not. This finding is consistent with observations discussed above regarding premotor cortex involvement in focal dystonia, and adds to the body of literature suggesting that premotor regions may be as important as M1 in focal dystonias. Other TMS studies supporting the application of TMS to address abnormal premotor function in focal dystonias are discussed below in “*Using imaging to evaluate and guide treatment of dystonia”*.

## USING IMAGING TO EVALUATE AND GUIDE TREATMENT OF DYSTONIA:

### Using Imaging to Evaluate Treatment Effectiveness

One of the most practically useful applications of imaging in focal dystonia is its use to evaluate patients before and after treatment to determine if (and how) clinical changes are exerted by the treatment. This is not only useful in showing that changes are exerted centrally, but also because it can help to map out the mechanisms by which the treatment is effective. Studies such as this were conducted evaluating neural changes in response to botulinum toxin (BTX) as early as 1997 [29]. Both functional (positron emission tomography or PET and fMRI) and structural measures have been used to evaluate neural changes in response to BTX [17, 23, 29, 110, 111]. These studies show widespread changes in some brain regions, but lack of change in other regions showing abnormalities in dystonia.

Interestingly, functional studies of BTX treatment seem more likely to show changes in premotor and/or basal ganglia regions than in M1 and primary somatosensory cortical regions [23, 29, 110], supporting the potential importance of premotor regions to dystonia discussed above. Moreover, this suggests that the premotor regions (possibly along with the basal ganglia) may be the source of abnormal function that feeds plastic changes in primary motor and sensory cortices, suggesting premotor function is the driving force that remaps output regions that actually implement output to the muscles. Such plasticity formed downstream of the “driver” of a neurological abnormality is challenging in that fixing the pathogenesis of dystonia will not necessary target all of the resulting pathophysiology. The slow timecourse of symptom improvement in DBS [112] is consistent with this, but does suggest that fixing the generator of the problem may not quickly or entirely reverse plasticity that has taken place downstream.

Imaging has also been used to evaluate the effects of surgical treatment of focal dystonia. A recent study by Lalli and colleagues [24] used F-fluoro deoxyglucose PET to show that epidural premotor cortical stimulation in two forms of primary focal dystonias (cervical or upper limb dystonia) led to a metabolic normalization in bilateral sensorimotor regions in these patients, along with symptom improvement (see Fig. (**3C**)).

Finally, magnetoencephalography (MEG) has been used to evaluate behavioral interventions to treat focal task-specific dystonias. One study showed that patients with writer’s cramp who underwent immobilization rehabilitation therapy, the size of the hand representation using MEG was closer to the size of this field in healthy subjects than to untreated patients, and this finding was specific to the hemisphere of the dystonic hand [113]. 

### Using Imaging to Select and Guide Treatment

Perhaps the most exciting area developing in the applications of imaging to the field of dystonia is in its potential use to predict optimal treatment for a given patient and/or to anatomically guide treatment. Given the extent of circuitry involved in dystonia, and number of potential causes, using imaging as a tool to develop biomarkers will be particularly important for sorting out different subtypes of pathophysiology [114, 115]. 

While standard structural MRI has long been used to guide brain surgery, more recently, diffusion tensor imaging has also begun to be used to target implantation of deep brain stimulation (DBS) electrodes directly in a specific white matter pathway (the dentate-rubro-thalamic tract, see Fig. (**5**); [116]). Such a method of stimulation may allow for much more directed targeting of stimulation to the affected neurons, while avoiding stimulation to other neurons which potentially produce side effects [116]. While this study was conducted in a patient with myoclonus dystonia to treat a tremor, and DBS is less commonly used in focal dystonias, the ability to more directly target the specific pathway of choice may, in the future, lead to increased ability to treat focal dystonias with this technique.

### Using TMS in Treatment of Focal Dystonias

Because TMS is thought to produce neurotrophic effects [117, 118], and has been shown to produce changes in the brain with repetition in dystonia, repetitive stimulation protocols (rTMS) have the potential to test properties of brain plasticity, and to exert therapeutic effects. Given that many forms of dystonia, particularly task-specific dystonias, are thought to include maladaptive plasticity as a factor (either endophenotypic or environmental), this paradigm is optimal for evaluating where and how plasticity is abnormal in dystonia [25, 119, 120]. Given that the plasticity induced by TMS has been shown to differ in patients with focal hand dystonia [121], determining what will work best for treatment will take some development. However, significant progress has already been made in this direction, and there is convergence with the fMRI findings discussed above, given it appears premotor stimulation is particularly effective in treating some focal dystonias [25, 26, 108, 109]. 

## SUMMARY

Brain imaging techniques have proven invaluable in identifying clues and testing hypotheses about the pathophysiology of focal dystonias. Recently, there has been mounting evidence about the importance of premotor regions and the cerebellum, network abnormalities, altered sensorimotor interactions, and altered inhibitory mechanisms in focal dystonias. These observations are convergently consistent with a hypothesis that dystonia is a disorder of postural control and stabilization mechanisms in the brain, which can be tested more directly in future studies evaluating these mechanisms. Data from TMS studies supporting decreased inhibition and/or increased excitation in the brain in focal dystonia may reflect a general etiologic principle by which these posture/stabilization mechanisms are abnormally amplified, although multiple potential etiologies likely underlie this general principle. From a practical perspective, imaging and TMS can be used to test effectiveness and locus of treatment impact on the brain, as well as to visually or etiologically guide treatment. In the future, it is hoped that this knowledge will aid in the application of imaging to individualize treatment of focal dystonias. 

## SOURCES OF FUNDING

The MGH Mood and Motor Control Laboratory is supported by a grant from the National Institute of Neurological Disorders and Stroke (grant number R01NS052368 to A.J.B.) and a grant from the Dystonia Medical Research Foundation to A.J.B. 

## Figures and Tables

**Fig. (1):**
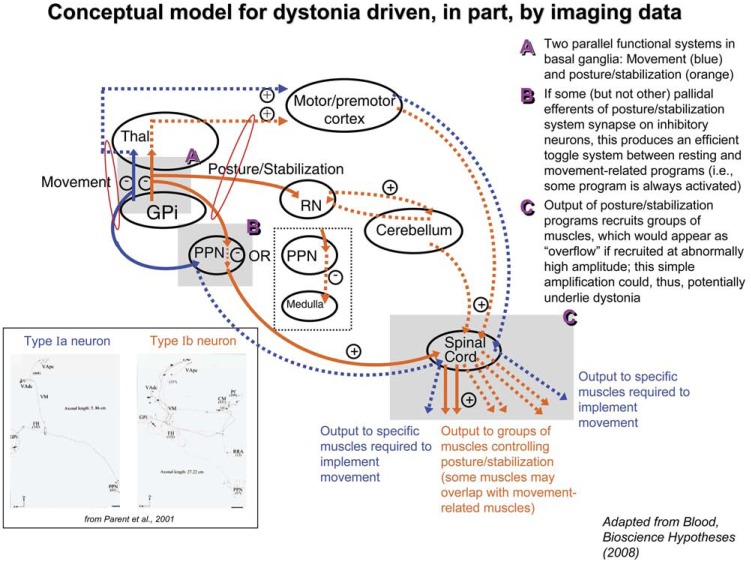
Conceptual model for dystonia and motor control driven by a combination of imaging data, motor circuitry cytoarchitecture, and clinical features of dystonia. This model builds on previous models and proposes three key new features that help to provide a more explicit mechanism for dystonia; these features are highlighted in gray shaded areas, **A**, **B**, and **C**, and are discussed in more detail in [15]. The model argues that dystonia is stereotyped because it reflects excessive amplification of a normal brain functional system. It also argues dystonia can be qualitatively heterogeneous because that functional system has several qualitatively different subcomponents. The proposed toggle system between activation of rest versus movement-related posture/stabilization programs would not only provide a highly efficient mechanism for coordinating across different postural subcomponents with a single neuron, but also offers a potential explanation why dystonia can be observed in both “low” and “high” dopamine states, such as in peak and end dose dystonias in Parkinson’s Disease [122]. The circuitry diagram here is shown in the “rest” or “tonic” dopamine state, and would be reversed during the “activated” or “phasic” state. Blue=movement-related circuitry; Orange=posture/stabilization-related circuitry. Solid lines indicate neuron is activated; dotted lines indicate neuron is inhibited. Plus and minus signs indicate whether a given neuron has an excitatory or inhibitory effect on its efferents (where multiple neuron types are present in a pathway, such as cerebellar output, no specific sign is indicated). GPi: internal globus pallidus; RN: red nucleus; PPN: pedunculopontine nucleus; Thal: thalamus.

**Fig. (2):**
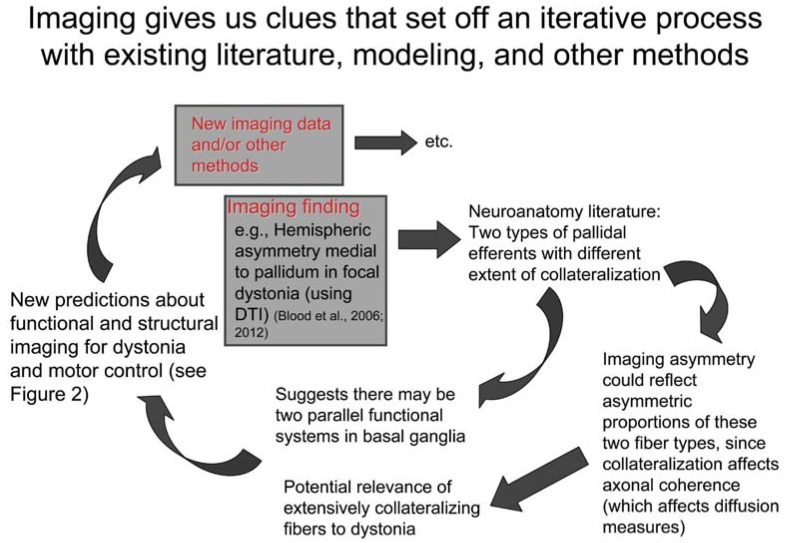
Schematic diagram of how imaging can be a hypothesis-generating tool that gives us clues about systems neuroscience and potential etiology of abnormalities in dystonia.

**Fig. (3):**
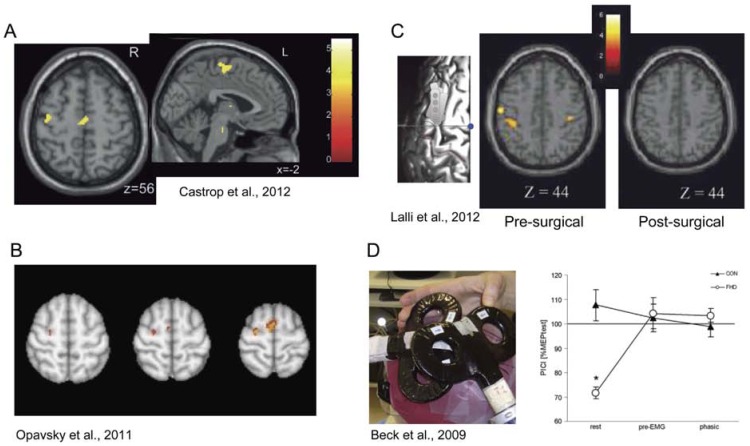
Both premotor abnormalities and treatment-related effects have been observed across several forms of focal dystonia. (**A**) Reduced activity in SMA and dorsal premotor cortex during movement imagination in writer’s cramp patients relative to healthy controls [21]. (**B**) Reduced ipsilateral dorsal premotor and supplementary motor area (SMA) activation in cervical dystonia patients after (versus before) treatment with botulinum toxin (BTX) [23]. (**C**) Epidural premotor stimulation in cervical dystonia/upper limb dystonia patients led to normalization of elevated glucose metabolism in dorsal premotor and SMA regions [24]. (**D**) A transcranial magnetic stimulation (TMS) conditioning pulse over left dorsal premotor cortex led to premotor-motor inhibition at rest in focal hand dystonia patients, but not controls. This inhibition was absent during movement in patients, suggesting premotor cortex may play some role in abnormal primary motor cortex surround inhibition during movement in these patients [108]. The y-axis depicts premotor intracortical inhibition (the ratio between the conditioned and unconditioned motor evoked potential [MEP]), and the x-axis shows the conditions: rest, motor preparation (pre-electromyography [pre-EMG]), and movement (phasic).

**Fig. (4):**
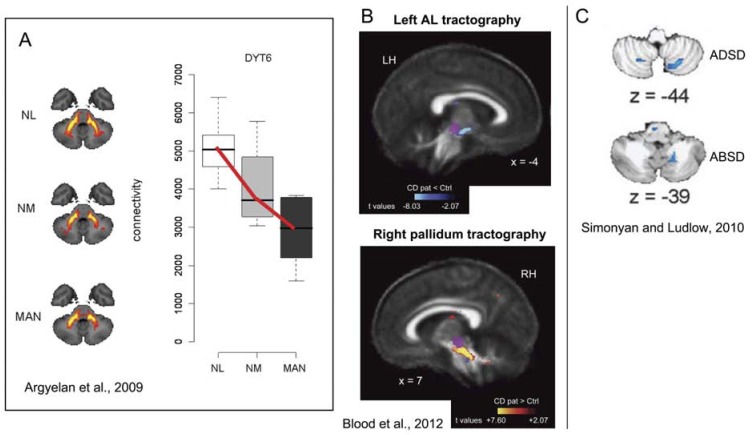
Both functional and structural abnormalities have been shown in the cerebellum across several forms of focal dystonia. (**A**) Individuals with the DYT6 dystonia mutation exhibited reduced tractography through the major outflow pathway of the cerebellum relative to healthy controls, and there was a group-wise trend in which manifesting carriers exhibited greater reductions than those who did not manifest [52]. Colorized images of group differences are shown here for combined DYT1 and DYT6 data; the box and whisker plot is shown for DYT6 patients only. (**B**) Cervical dystonia patients exhibited reduced and increased probabilistic tractography from the left ansa lenticularis (AL) and right pallidum, respectively; the regions of difference intersect with the red nucleus (in purple), which is a major relay nucleus of the cerebellum [55]. (**C**) Both adductor (ADSD) and abductor (ABSD) forms of spasmodic dysphonia showed increased activation in the cerebellum during symptomatic syllable production, relative to controls [57].

**Fig. (5):**
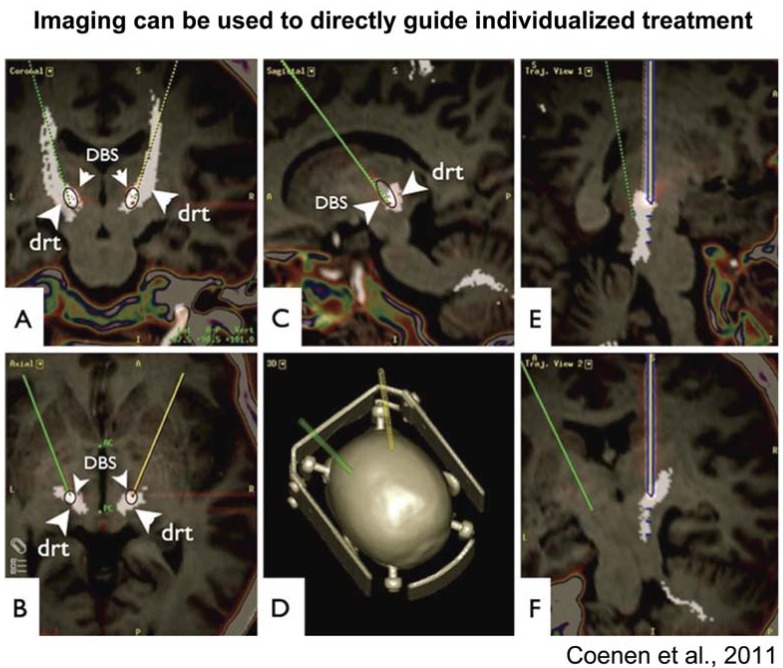
Diffusion tensor imaging (DTI) tractography can be used for individualized placement of deep brain stimulation electrodes in white matter tracts. The figure shown here illustrates how this method was used for electrode placement in the dentato-rubro-thalamic tract in a therapy-refractory tremor patient (from [116]).
